# Fibrodysplasia ossificans progressiva in Hong Kong—A case report series

**DOI:** 10.3389/fped.2023.1152731

**Published:** 2023-04-25

**Authors:** Joshua Chun Ki Chan, Evelyn Eugenie Kuong, Joyce Pui Kwan Chan, Ho Ming Luk, Jasmine Lee Fong Fung, Joanna Yuet-ling Tung, Brian Hon Yin Chung

**Affiliations:** ^1^ Department of Paediatrics and Adolescent Medicine, Queen Mary Hospital, Hong Kong, Hong Kong SAR, China; ^2^ Skeletal Dysplasia Joint Clinic, Hong Kong Children's Hospital, Hong Kong, Hong Kong SAR, China; ^3^ Department of Orthopaedics and Traumatology, Duchess of Kent Children's Hospital, Hong Kong, Hong Kong SAR, China; ^4^ Department of Radiology, Hong Kong Children's Hospital, Hong Kong, Hong Kong SAR, China; ^5^ Clinical Genetics Service Unit, Hong Kong Children's Hospital, Hong Kong, Hong Kong SAR, China; ^6^ Department of Paediatrics and Adolescent Medicine, School of Clinical Medicine, LKS Faculty of Medicine, University of Hong Kong, Hong Kong, Hong Kong SAR, China; ^7^ Department of Paediatrics and Adolescent Medicine, Hong Kong Children's Hospital, Hong Kong, Hong Kong SAR, China

**Keywords:** case report, fibrodysplasia ossificans progressiva, *ACVR*1, Hong Kong, FOP

## Abstract

Fibrodysplasia ossificans progressiva (FOP) is an ultra-rare condition. The diagnosis could be challenging due to its rarity and non-specific presenting symptoms. However, early diagnosis and appropriate management help in preserving patients' function and quality of life. Herein, we report the diagnostic journeys and clinical courses of 8 patients with FOP in Hong Kong and illustrate the challenges involved.

## Introduction

Fibrodysplasia ossificans progressiva (FOP) is an ultra-rare genetic disease caused by mutations in the *ACVR1* gene. The classical features include congenital big toe malformations, heterotopic ossifications resulting from inflammation or trauma to soft tissues, and progressive limitation in ranges of joints motion (1). The initial presentation could be non-specific and diagnosis could be challenging. However, early diagnosis guides management strategies which could halt disease progression, preserve function, and reduce morbidity.

We describe herein the diagnostic journeys and clinical courses of eight patients with FOP in Hong Kong. Patients were identified using the database of the local clinical genetic services, including the University of Hong Kong and Clinical Genetics Service, Department of Health.

Clinical information was retrieved from the Electronic Patient Record of Hong Kong Hospital Authority. To assess and compare the clinical severity of the disease objectively, the Cumulative Analogue Joint Involvement Scale (CAJIS) score was used (2). It grades 15 different joints over the body according to the limitation in range of motion, with a score of ‘0’ if the joint is not affected, a score of ‘1’ if the joint is affected with limitation in range of motion, and ‘2’ if the joint is functionally ankylosed (2). The maximum score is 30.

Consent for publication had been obtained from all patients.

## Case description

Eight patients were included in this review, namely, three boys and five girls. All patients carried the classical pathogenic *ACVR1*:c.617G > A mutation, and all occurred *de novo*. The mean age at presentation was 4.1 years (range: 0–13 years), and the mean age at diagnosis was 7.9 years (range: 3–15 years). The average time from initial presentation to diagnosis was 3.8 years (range: 0–7 years). Their mean age at last follow-up was 17.3 years (range: 6–28 years). The commonest initial presentation was joint stiffness, which was observed in half of the patients; followed by recurrent, waxing and waning masses over the upper body. Various forms of congenital big toe malformations were present in all patients. Five out of the eight patients (62.5%) had received a prior wrong diagnosis, and invasive procedures had been performed on two of them, including surgical excision and biopsy of the masses. Upon the latest follow-up, six out of eight patients (75.0%) suffered from comorbidities other than joint stiffness. These included conductive hearing loss (*n* = 4, 50.0%), temporomandibular joint ankylosis (*n* = 3, 37.5%), and restrictive lung disease (*n* = 2, 25.0%). Most patients required some form of assistance in activities of daily living, three (37.5%) were wheelchair dependent and only two (25.0%) were able to walk independently with minimal overt abnormalities.

Patient 1 presented at 5 years old with multiple joint stiffness. The initial radiological diagnosis was diaphyseal dysplasia. He developed conductive hearing loss at 8 years old. At 12 years old, he developed a large mass over the back following a back strain. The genetic diagnosis of FOP was made after reviewing the clinical history by an overseas expert. Subsequently the patient experienced 3–4 flares per year, but he was non-compliant to steroids during the flares. There was progressive limitation in the ranges of motion of most joints, and he became wheelchair-dependent and activity of daily living (ADL)-dependent by 16 years old with generalized joint ankylosis. He was unable to sit and had to stand or lie flat even during urination and defecation.

Patient 2 presented at 1 year old with recurrent, waxing and waning scalp masses. At 7 years old, she developed a persistent painful mass over the back following a blunt injury, which was noted to be heterotopic ossification by radiographs. Genetic testing confirmed the diagnosis of FOP. She developed frequent flares and was treated with steroids, montelukast and non-steroidal anti-inflammatory drugs (NSAIDs) during the acute flares. By the age of 9 years, she had temporomandibular joint ankylosis and dental caries. She developed obstructive sleep apnea at 11 years old and had conductive hearing loss by 12 years. By adolescence, she had generalized joint ankyloses and contractures and was wheelchair-bound.

Patient 3 presented at 4 years old with neck stiffness, with radiographs showing multi-level cervical spinal fusion. At 6 years old, she developed a right shin nodule which was excised. At 9 years old, she developed another mass over the left knee following a sprain, which was excised but recurred at the operation site. She was referred to Clinical Genetics Service at 11 years old, and the diagnosis of FOP was made. She developed conductive hearing loss at 13 years old. Subsequently, she experienced occasional flares which were treated with steroids and NSAIDs. By the age of 26 years, she had contractures over her shoulders and hip. She was mostly homebound but was able to walk with a stooped posture.

Patient 4 presented at birth with recurrent, waxing and waning masses over the head, neck, and back. At 3 years old, she received excisional biopsy for a central neck mass and was given the diagnosis of nodular fasciitis, and given a short period of sirolimus at 4 years old with no significant improvement. She developed heterotopic ossification over a fractured left radius following an injury at 5 years old, and the diagnosis was revised to FOP following genetic testing. By 8 years old, she had severe kyphoscoliosis with fixed contractures over the shoulders and elbows. She could walk unaided with a stoop posture and is ADL-independent at the moment.

Patient 5 presented at 13 years old with insidious and progressive neck and shoulder stiffness since pre-school, preceded by swellings which waxed and waned. She also had reduced jaw movement following an episode of oral ulcer. At 15 years old, she developed masses over her back which stiffened her back, and radiographs revealed heterotopic ossifications. Genetic testing confirmed the diagnosis of FOP. Subsequently, she developed ankyloses of the whole spine and temporomandibular joint, with stiffness in both shoulders and elbows. Her lower limbs were unaffected and she could walk unaided.

Patient 6 presented at 5 years old with reduced left hip movement. Radiographs also revealed bilateral hallux valgus with dysplastic metatarsal–phalangeal joint, and he was clinically diagnosed to have FOP, which was later genetically confirmed. He had conductive hearing loss at 13 years old. He developed infrequent flares which were treated with steroids and NSAIDs, with slow progressive joint contractures mainly affecting the upper body, but was able to walk unaided with a limp, and largely independent in activities of daily living.

Patient 7 presented at 5 years old with easy falling and stiffness over the neck, back, and shoulder. Radiographs revealed ectopic ossifications posterior to the spine, and genetic testing confirmed the diagnosis of FOP. She was given steroids and NSAIDs for flares, and she remained stable with activities of daily living minimally affected.

Patient 8 was noted to have bilateral hallux valgus at birth, and magnetic resonance imaging (MRI) showed dysplastic first metatarsals and proximal phalanx, with apparently fused metatarsal and proximal phalanx epiphyses. FOP testing was arranged based on radiological findings and confirmed the diagnosis. He was given steroids and NSAIDs to treat subsequent flares during childhood, with mostly full recovery in joint ranges of motion. Though he had bilateral acetabular dysplasia and multiple lower limb exostoses by 5 years old, he walked well unaided.

**Figure 1:**
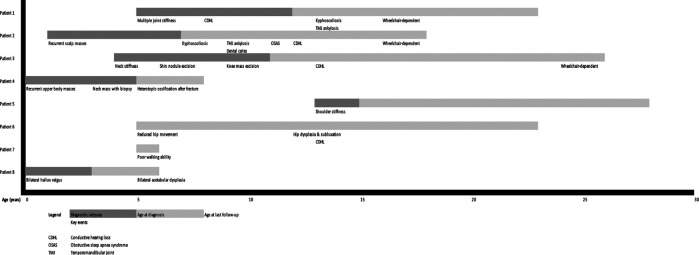
Timeline demonstrating the clinical courses of all eight patients with relation to age.

**Figure 2:**
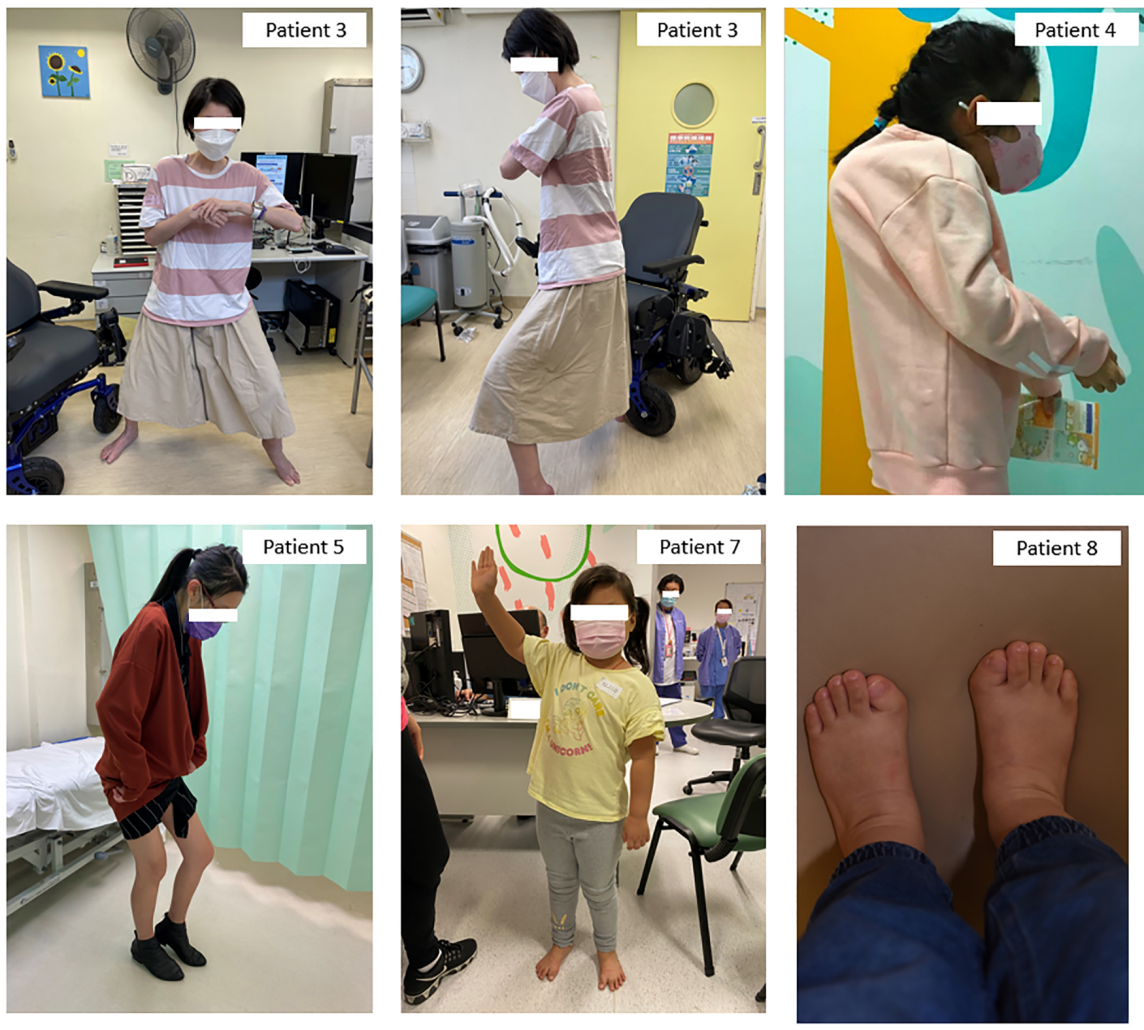
Clinical photos of Patients 3, 4, 5, 7, and 8. Patient 3: Bilateral shoulder and left elbow contractures with inability to raise hands above shoulder level, also bilateral hip and knee flexion contractures resulting in stooped posture even during walking, depends on powered wheelchair for mobility. Patient 4: Bilateral shoulder and elbow contractures and severe kyphoscoliosis with impending chin-on-chest deformity. Patient 5: Unaffected lower limbs but fully ankylosed spine from cervical to lumbar in kyphotic position, needs to stand with hips and knees flexed to maintain balance. Patient 7: Limited right shoulder abduction with decreased range of motion and other joints are minimally affected otherwise. Patient 8: Characteristic big toe deformity with hallux valgus.

**Figure 3:**
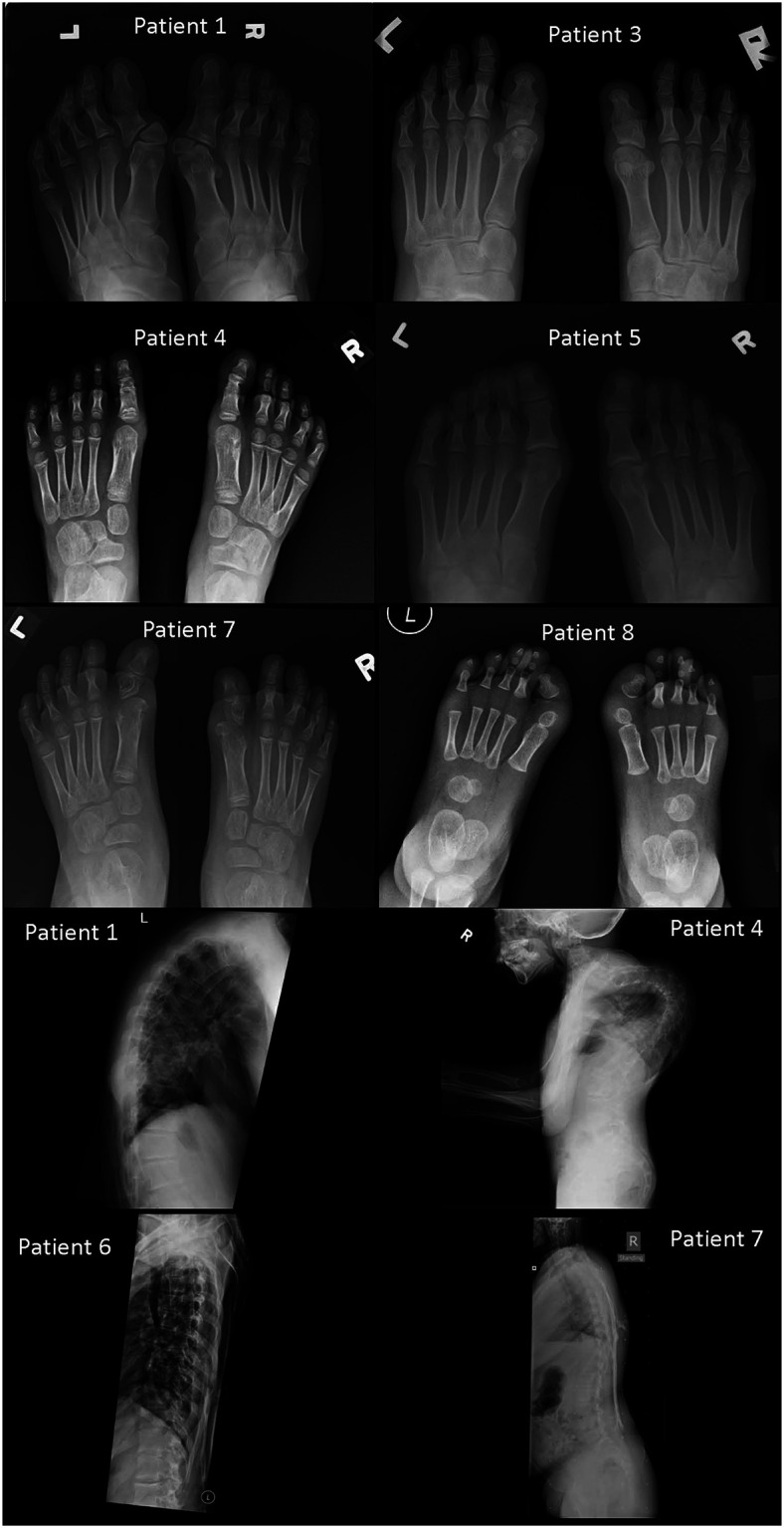
Foot radiographs from Patients 1, 3, 4, 5, 7, and 8, and spine radiographs from Patients 1, 4, 6, and 7 (foot radiographs). Patient 1: Deformed heads of bilateral first metatarsals and proximal phalanges with hallux valgus. Fused proximal and distal phalanges of both big toes. Patient 3: Deformed heads of bilateral first metatarsals and phalanges of bilateral big toes. They are hypoplastic and short with fusion of proximal and distal phalanges of both big toes. Patient 4: Tri-phalangeal big toes on both sides with fusion of the proximal and middle phalanges. Subtle hallux valgus. Patient 5: Subtle hallux valgus. Patient 7: Dysplastic heads of bilateral first metatarsals and proximal phalanges of both big toes. Right big toe appears short. Patient 8: Bilateral heads of first metatarsals appear dysplastic with short hypoplastic bilateral big toes with single phalanx. Bilateral hallux valgus (spine radiographs) Patient 1: Ossifications of the paravertebral soft tissue demonstrated on the lateral view with a kyphotic spinal curvature. Patient 4: Extreme kyphoscoliosis of the whole spine with segments of cervical spine fusion and impending chin-on-chest deformity. Patient 6: Relative preservation of normal thoracic spinal alignment with no major heterotopic ossifications. This spinal radiograph is taken at similar age as that of Patient 1. Patient 7: Paraspinal soft tissue ossifications extending from upper back down to upper sacral level with relatively preserved vertical spinal alignment. This spinal radiograph is taken at similar age as that of Patient 4.

The clinical characteristics of these eight patients were summarized in Table 1 and their clinical courses were compared with each other in Figure 1. Figures 2, 3 show illustrative clinical photos and radiographic images for the patients.

**Table 1:** Summary of clinical characteristics of all eight patients. The CAJIS scores are estimated from the latest clinical notes.

	Patient 1	Patient 2	Patient 3	Patient 4	Patient 5	Patient 6	Patient 7	Patient 8
Sex	M	F	F	F	F	M	F	M
Age at initial presentation (years)	5	1	4	Birth	13	5	5	Birth
Initial presentation	Multiple joint stiffness	Recurrent masses over scalp	Stiff neck	Recurrent masses over head/neck/back	Stiff shoulders	Reduced hip movement	Poor walking ability	Bilateral toe deformity
Age at diagnosis (years)	12	7	11	5	15	5	5	3
Time from presentation to diagnosis (years)	7	6	7	5	2	–	–	3
Big toe malformation	Deformed heads of bilateral first metatarsals and proximal phalanges Fused big toe proximal and distal phalanges Hallux valgus	Deformed bilateral first metatarsals and proximal phalanges Hallux valgus	Deformed heads of bilateral first metatarsals and phalanges Hypoplastic and short big toes with fused proximal and distal phalanges	Tri-phalangeal big toes with fused proximal and middle phalanges Subtle hallux valgus	Subtle hallux valgus	Oblique metatarsal-phalangeal joint Hallux valgus	Dysplastic heads of bilateral first metatarsals and proximal Right big toe appears short	Bilateral heads of first metatarsals appear dysplastic Short hypoplastic big toes with single phalanx Hallux valgus
Estimated CAJIS score at diagnosis	3	7	2	9	2	2	5	0
Age at last follow-up (years)	23	18	26	8	28	23	6	6
Estimated CAJIS score at last follow-up	24	10	9	9	13	10	5	0
Mobility at last follow-up	Wheelchair-dependent Carer-dependent	Wheelchair-dependent Assistance with some ADLs	Wheelchair-dependent Assistance with some ADLs	Walk in stooped posture Carer-dependent	Walk with stick	Walk with limp	Independent	Independent
Comorbidities	Hearing loss TMJ ankylosis Restrictive lung disease	Hearing loss TMJ ankylosis Dental caries OSAS	Hearing loss	Restrictive lung disease	TMJ ankylosis	Hearing loss	Nil	Nil

ADL, activities of daily living; CAJIS, Cumulative Analogue Joint Involvement Scale; TMJ, temporomandibular joint; OSAS, obstructive sleep apnoea syndrome.

## Discussion

We described the clinical course of eight patients with genetically confirmed FOP, including their initial presentation, diagnostic odyssey, and latest clinical outcome. In Hong Kong, the genetic diagnosis of FOP is mostly provided by Clinical Genetics Service of the Department of Health. Therefore, we believed that we have covered all the genetically confirmed cases of FOP in Hong Kong.

FOP is an extremely rare condition. There are only around 800 cases reported worldwide, and the estimated prevalence was quoted as 0.6–1.36 per million in previous literature (3–5). Within a population of 7.5 million in Hong Kong (6), a total number of 8 cases is close to the expected number calculated from the quoted prevalence. Nevertheless, it is possible that there are more patients who had never been referred to Clinical Genetics Service and never received a correct diagnosis. The ‘classical’ *ACVR1*:c.617G > A mutation was postulated to contribute to the majority of all FOP cases (7), and this mutation was identified in all of our reported patients.

Initial presentations were variable. The most common initial presentation was joint stiffness or reduced ranges of motion, followed by recurrent masses mostly over the upper body, while one patient presented with a non-specific complaint of easy falling. The first specialty consulted included orthopaedics, paediatrics, or paediatric surgery. The mean time from first presentation to receiving a correct, final diagnosis was 3.8 years, which was similar to the reported international cohorts (8). Radiological abnormalities of the big toes were seen in all patients, but these deformities had a wide spectrum of severity, ranging from the classical bilateral hallux deformity to only subtle changes that were not clinically apparent until the diagnosis of FOP was suspected and radiograph was closely reviewed. Indeed, this important hint was not picked up clinically in all except one case in their initial presentation. Extensive investigations were performed in half of the patients, including computed tomography, magnetic resonance imaging, and excisional biopsy. However, these results often created more confusion and distractions, rather than helping to reach the diagnosis. This is because most investigatory findings would only reveal non-specific features of inflammation before the formation of heterotopic ossification. In Patient 4, for example, excisional biopsy of the lesion was interpreted as nodular fasciitis, which was thought to be her ultimate diagnosis, yet indeed further lengthened the diagnostic odyssey.

Similar to most other cohorts (7–9), most patients followed a slowly progressive, deteriorating clinical course, which was progressive with age, and aggravated by trauma. In addition, invasive interventions were also associated with worse clinical outcome, as demonstrated in Patients 3 and 4 who both underwent surgical procedures prior to the diagnosis of FOP. Their disease and functional statuses were significantly worse compared with other patients at similar ages. In contrast, Patients 7 and 8, who were both of similar age as Patient 4 at last follow-up, received the diagnosis relatively earlier and did not undergo any invasive procedures. They were both walking unaided with only mild joint stiffness and minimal disruption to activities of daily living.

Our findings are consistent with previous reports that misdiagnosis and delayed diagnosis are common in FOP, attributable to the non-specific presentation and lack of awareness among general attending physicians, and unnecessary investigations causing long-term adverse effect on morbidity and quality of life (10). Although FOP is ultra-rare, the congenital malformations of the big toe are characteristic, easily identifiable and serve as important hints towards the correct diagnosis. There is a need to increase the awareness and knowledge towards this condition among doctors, in particular paediatricians and orthopaedic surgeons who often are the first doctors to be encountered by the patients. This would improve the ability to accurately identify this condition and avoid unnecessary, harmful, and invasive investigations or procedures.

Although all patients shared the same classical mutation, which was previously described to have a typical and rather predictable clinical course compared with other mutations (9), there was still significant variability in their phenotypes. Patient 5, for example, did not present until 13 years old with insidious shoulder stiffness followed by a slow pace of clinical deterioration, while other patients started to experience marked joint stiffness or even episodes of flares in early childhood and already had significant morbidity before entering adolescence. Some patients presented with recurrent lumps and bumps during infancy while others did not. Although most patients had an identifiable hallux deformity, the degree varied, and some were valgus instead of the classical varus. It is also possible that other genetic or epigenetic modifiers are present that affect the phenotype. Last but not the least, it must be emphasised again that the clinical severity could largely be affected by prior surgical interventions, which were indeed unnecessary.

FOP is associated with significant morbidity and increased mortality. The average lifespan from the international patient registry was estimated to be 56 years old. Cardiopulmonary failure resulting from thoracic insufficiency or pneumonia was the most common cause of death (11). Another rare but debilitating and potentially life-threatening complication is the chin-on-chest deformity—it develops with orthotopic fusion of multiple levels of cervical spine, particularly in the child as the neck continues to grow while being tethered to the spine and is associated with high mortality rate and also difficult to correct surgically (12). Patient 4 in our series has severe kyphoscoliosis at a very young age, and she would likely develop this problem in the future, as demonstrated in Figure 2.

There has been no specific treatment for FOP, and the management strategies are mainly conservative, through lifestyle modification to minimize risks of trauma including fall prevention, and treatment of flares using steroids and non-steroidal anti-inflammatory drugs (NSAIDs) to reduce inflammation and alleviate symptoms (13). This approach has been implemented in all of our patients. Patients suffered from a range of significant morbidities and would benefit from an individualized multidisciplinary approach to preserve quality of life and prevent further deterioration, such as gentle, active stretching exercise instructed by physiotherapists to reduce pain and contractures, and regular dental check-up to minimise dental procedures from precipitating temporomandibular joint ankyloses. Mast cell has also been proposed to play a vital role in the pathogenesis of FOP, in particular progressive injury-induced heterotopic ossifications (14). The leukotriene receptor antagonist Montelukast inhibits mast cell activation and blocks inflammatory mediators and has also been used in some of our patients. It is recommended as a Class II medication in the treatment for FOP, is generally well tolerated but has possible associations with behaviour and mood changes including risk of suicidal thoughts (13). With further research and improved understanding of the underlying molecular pathomechanism of FOP, targeted drugs are under development and clinical trials are ongoing to seek a definitive cure (15). More notably, the oral retinoic acid receptor γ (RARγ) agonist, Palovarotene, is the first drug to have been approved as treatment for FOP in Canada in January 2022 (16). It has been demonstrated in Phase 2 clinical trials to be able to reduce the volume of heterotopic ossifications in flare-ups by 70% compared with placebo (17) and is currently undergoing Phase 3 clinical trials. Another promising agent is the ACVR1/ALK2 receptor ligand monoclonal antibody Garetosmab, which had been shown to reduce flare-ups by 50% compared with placebo (18).

There are several limitations of our study. Although we examined a population-based cohort, the sample size remained small, undermining the statistical power of subgroup analyses. There is also lack of a territory-wide treatment protocol with uniform and systematic evaluation for each patient at specific time points. In the past, there was no designated centre in Hong Kong for FOP, and different patients were managed by different specialties of doctors. With the recent establishment of the Hong Kong Children's Hospital, newly diagnosed cases could be referred to a dedicated, multidisciplinary team, consisting of clinical geneticists, paediatric endocrinologists, orthopaedic surgeons, clinical radiologists, dental surgeons, physiotherapists, occupational therapists, and clinical psychologists. This centralized and one-stop model not only allows highly focused and specialized expertise to provide care for patients with this ultra-rare disease where most primary care centres may have little experience with, but also combines services of various subspecialists to incorporate the optimal management plan for the patients. At the same time, this enables patients to be seen by multiple doctors during the same session, reducing the number of hospital visits needed for them, who often suffer from reduced mobility. This current cohort could serve as a local benchmark for future comparison.

## Conclusions

FOP is an ultra-rare disease, and initial presentations are usually non-specific such as joint stiffness or masses over the body, which can be confused with other diseases. This leads to unnecessary invasive procedures, which aggravates the condition. Early diagnosis and appropriate management helps alter disease course and preserve body function and quality of life. Coordinated, multidisciplinary management with close collaboration and communication among different subspecialties is the key of success in managing these patients with multiple needs. Steroids and NSAIDs help alleviate symptoms during flares. Research and drug trials on specific, curative treatment targeting the underlying molecular pathway are underway, and it is hoped that the disease course of these patients would be modified or even transformed in the near future.

## Data Availability

The original contributions presented in the study are included in the article/Supplementary Material. Further inquiries can be directed to the corresponding author.
